# Resistance of R-Ras knockout mice to skin tumour induction

**DOI:** 10.1038/srep11663

**Published:** 2015-07-02

**Authors:** Ulrike May, Stuart Prince, Maria Vähätupa, Anni M. Laitinen, Katriina Nieminen, Hannele Uusitalo-Järvinen, Tero A. H. Järvinen

**Affiliations:** 1 School of Medicine, Department of Anatomy and Cell Biology, University of Tampere, Tampere, Finland; 2 Department of Ophthalmology, Tampere University Hospital, Tampere, Finland; 3 Department of Orthopedics & Traumatology, Tampere University Hospital, Tampere, Finland

## Abstract

The *R-ras* gene encodes a small GTPase that is a member of the Ras family. Despite close sequence similarities, R-Ras is functionally distinct from the prototypic Ras proteins; no transformative activity and no activating mutations of R-Ras in human malignancies have been reported for it. R-Ras activity appears inhibitory towards tumour proliferation and invasion, and to promote cellular quiescence. Contrary to this, using mice with a deletion of the *R-ras* gene, we found that R-Ras facilitates DMBA/TPA-induced skin tumour induction. The tumours appeared in wild-type (WT) mice on average 6 weeks earlier than in R-Ras knockout (R-Ras KO) mice. WT mice developed almost 6 times more tumours than R-Ras KO mice. Despite strong R-Ras protein expression in the dermal blood vessels, no R-Ras could be detected in the epidermis from where the tumours arose. The DMBA/TPA skin tumourigenesis-model is highly dependent upon inflammation, and we found a greatly attenuated skin inflammatory response to DMBA/TPA-treatment in the R-Ras KO mice in the context of leukocyte infiltration and proinflammatory cytokine expression. Thus, these data suggest that despite its characterised role in promoting cellular quiescence, R-Ras is pro-tumourigenic in the DMBA/TPA tumour model and important for the inflammatory response to DMBA/TPA treatment.

R-Ras is a small GTPase of the Ras family of known oncogenes that was originally identified as a close homolog of oncogenic H-Ras^1^. Despite its close structural similarity to other members of the Ras-family, the function of R-Ras is distinct from the prototypic Ras proteins (K-, H-, N-Ras)^2^. Whereas a single amino acid mutation can convert other members of the Ras family into oncogenes, the equivalent mutations in R-Ras did not induce transformative activity^3^. Neither have there been any activating mutations reported for R-Ras in human malignancies, whereas such mutations in other Ras members are considered a common hallmark in a large number of cancers^4^. To further highlight the apparent non-oncogenic nature of R-Ras, it was recently shown that R-Ras actually inhibits all landmark features of cancer; proliferation, migration and cell cycle progression in breast cancer cells *in vitro*^5^.

The distinct role of R-Ras among the Ras family also extends to cell signaling^2^. Unlike K- and H-Ras, R-Ras does not activate Raf-1 and RalGDS^6^. R-Ras activity elevates the affinity and avidity of integrins and enhances cell adhesion to the extracellular matrix^7^, whereas these effects are antagonised by H-Ras-Raf signaling^8^. Furthermore, it was recently shown that endothelial R-Ras inhibits vascular cell proliferation and tumour invasion and promotes vascular quiescence and integrity during tumour angiogenesis and in response to arterial injury^9^,^10^.

Despite a previous study indicating that R-Ras is not expressed in normal epithelial tissues^10^, aberrant over-expression of R-Ras has been reported to take place in cancers of epithelial origin, e.g. breast^5^, gastric^11^ and cervical cancer^12^. Thus, we assumed that carcinogen-initiated tumour formation could induce R-Ras expression and we decided to explore the role of R-Ras in a skin epidermal carcinogenesis model (two-stage DMBA/TPA model) in wild-type (WT) and R-Ras knockout mice (R-Ras KO).

## Results

### Dermal R-Ras plays an important role in skin tumour induction and size

To investigate the role of R-Ras in skin tumour formation, we treated the back skin of adult mice deficient for *R-ras* gene expression (R-Ras KO^10^,) and wild-type mice (WT, as control) once with a local application of the mutagen DMBA, and then repeatedly with the growth-promoting histone deacetylase inhibitor TPA, twice weekly for a period of 19 weeks. This treatment induces papillomas derived from the interfollicular epidermis^13^.

**Figure 1:**
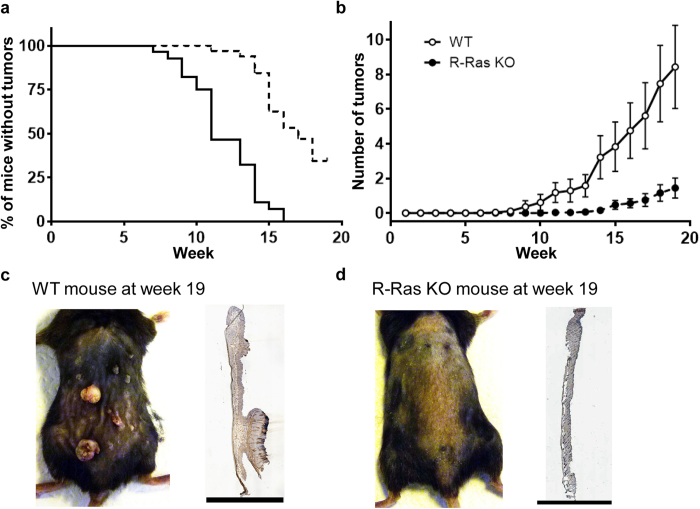
R-Ras is crucial for skin tumour formation. Wild-type (WT, solid line) and R-Ras knockout (R-Ras KO, dashed line) mice were subjected to DMBA/TPA-induced skin carcinogenesis as described in the methods section. Two individual trials were performed, both trials yielded a very similar outcome, and the data (WT: n = 13 and 15; R-Ras KO: n = 18 and 14) were combined. (**a**) The percentage of tumour-free animals at each time point is shown. Because the proportional hazards assumption appeared correct, a survival plot was generated and analysed via log-rank (Mantel-Cox) test, as described in methods. The data for the WT and R-Ras KO groups were highly significantly different (P < 0.0001). The median time to tumour onset in the WT mice was 11 weeks, whereas for the R-Ras KO mice it was 17 weeks. (**b**) The mean number of tumours per mouse at each time point is shown ± 95% confidence interval. The data were analysed using STATA 13.0 software as described in methods. Because the data was count data (not normally distributed), a non-linear regression model was used to compare the slopes of the data. Because the variances of tumour number in the R-Ras KO and WT mice were larger than the means of tumour number (i.e. over-dispersed), negative binomial regression was selected to analyse the data. The data from the two groups were highly significantly different (P < 0.001). The R-Ras KO mice had on average 3.2 × (95% CI 1.97, 5.21) fewer tumours than the WT mice. At the end of the trial, WT mice had on average 5.86 × more tumours than the R-Ras KO mice. (**c**) Representative photograph of a WT mouse at the end of the DMBA/TPA treatment trial, alongside a hematoxylin-eosin stained section of skin (the black bar represents 7 mm). The abundance of small and large tumours upon the skin can be clearly seen. (**d**) Representative photograph of an R-Ras KO mouse at the end of the DMBA/TPA treatment trial, alongside a hematoxylin-eosin stained section of skin (the black bar represents 6 mm). There are no visible tumours.

The first papillomas were observed in the WT mice 7 weeks after the beginning of the DMBA/TPA treatment, and after 16 weeks, all 28 of these mice had developed papillomas (Fig. 1a). The number of papillomas per WT mouse steadily increased reaching approximately 8 tumours per mouse after 19 weeks (Fig. 1b). Animals with a deletion of R-Ras, however, were very resistant to skin tumour induction (Fig. 1a–d). Tumours arose in R-Ras KO mice 6 weeks (median) later than in WT mice. Fifty three percent of the R-Ras KO mice had not developed even a single papilloma after 16 weeks of treatment, whereas all of the WT mice had. Only 11 out of the 32 R-Ras KO mice developed more than one papilloma during the study and we never observed large papillomas in R-Ras KO littermates as were seen frequently in WT animals (Fig. 1c,d). The few papillomas in R-Ras KO mice were too small (<2 mm diameter) to require angiogenesis for growth. Furthermore, we could detect almost no papillomas in histological samples from R-Ras KO mice, when different histological analyses (H&E, R-Ras, CD31, Ki67, apoptosis) were performed from the pre-set location of the back skin exposed to chemicals (Fig. 1d). During the course of experiments the tumours were incident in WT animals at a rate on average threefold greater than in R-Ras KO mice (negative binominal regression analysis: incidence rate ratio (IRR) = 3.2; 95% confidence interval (CI) 1.97, 5.21). At the end of the experiments (19 weeks after DMBA/TPA treatment), WT animals had on average almost 6 times more tumours than the R-Ras KO mice.

### Skin R-Ras expression is restricted to blood vessels in the dermis

**Figure 2:**
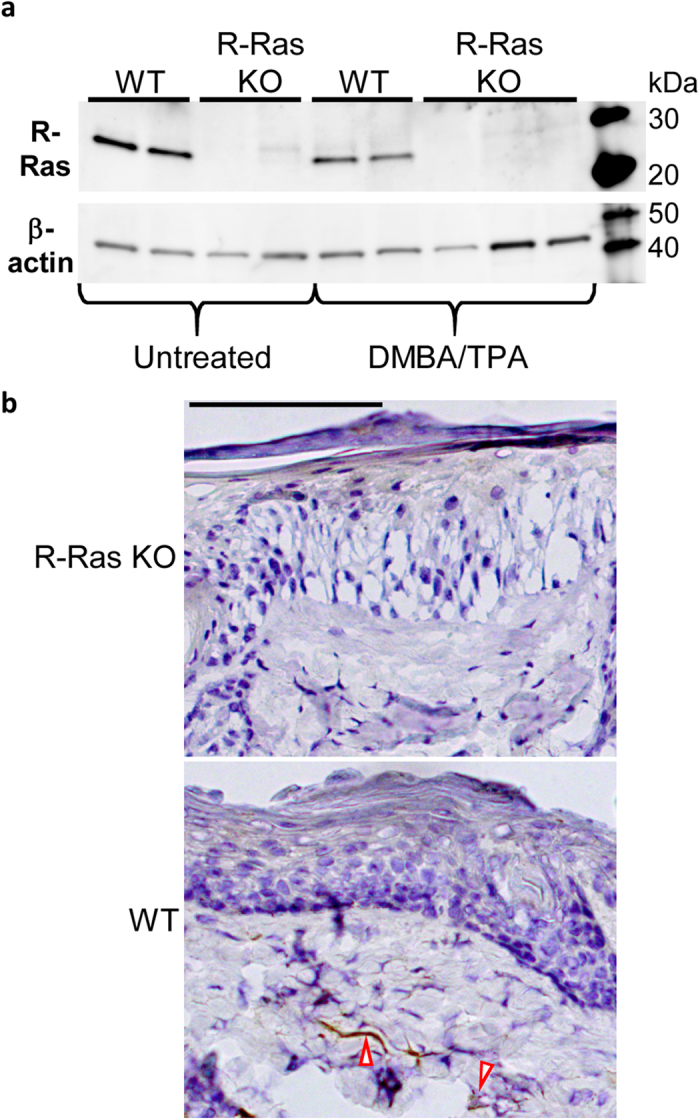
R-Ras is expressed exclusively in the blood vessels in the dermal part of the skin, but not in the epidermis. Both untreated and DMBA/TPA-treated skins were collected and either fixed for immunohistochemistry (IHC), or homogenised and lysed in RIPA buffer for western blot analysis, and R-Ras expression detected as described in the methods section. (**a**) Detection of R-Ras protein in Western blot analysis of skin lysates. The blot was stripped and reprobed with β-actin as loading control. The images displayed are cropped (full-length blots/gels are presented in Supplementary Figure S1). The WT mice were confirmed to express R-Ras in their skin, while the KO mice did not express R-Ras. The relative mean R-Ras protein expression was analysed by densitometry: WT untreated: 0.91 with 95% CI −0.21, 2.034; WT DMBA/TPA treated: 0.52 with 95% CI 0.47, 0.57. Cropped pictures are shown. (**b**) DMBA/TPA-treated WT skin (including tumours) and R-Ras KO skin was fixed with 4% paraformaldehyde, embedded in paraffin, and stained for R-Ras protein expression by IHC. Representative photographs of the results are shown. The R-Ras KO mice were confirmed not to express R-Ras at all. In the WT mice strong R-Ras expression can be seen in the dermal blood vessels (red outlined arrows), while some very occasional dermal cells (or infiltrating migratory cells) may also weakly express R-Ras. Epidermis is devoid of any R-Ras expression. The black bar represents 100 μm.

Strong R-Ras protein expression was detected by western blot analysis both in untreated and DMBA/TPA-treated skin of the WT mice (Fig. 2a). Interestingly, R-Ras protein levels seemed to slightly decrease in the WT following DMBA/TPA treatment (Fig. 2a). We also confirmed the total lack of R-Ras protein in the R-Ras KO mice both in untreated and DMBA/TPA-treated skin samples (Fig. 2a). We could not detect any R-Ras protein expression in the epidermal cell layer either in untreated or DMBA/TPA-treated WT mice (Fig. 2b). Blood vessels (as well as very rarely some occasional dermal cells) expressed R-Ras protein in the WT, but the epidermis (and tumours) remained negative for R-Ras expression (Fig. 2b).

### The resistance of R-Ras KO mice to skin tumourigenesis is not associated with decreased vascularisation

To understand the mechanism of the tumour-promoting function of R-Ras in the skin, we continued to analyse the whole skin by determining the epidermal and dermal thickening, and the amount of vasculature (angiogenesis) in the back skin of DMBA/TPA treated and untreated mice. All analyses were performed from the same part of the back skin in all animals to avoid any bias (such as selecting a plane of analyses to go through tumour).

In untreated mice, loss of R-Ras had minor effects on epidermal and dermal thicknesses, as the R-Ras KO mice had a slightly thinner epidermis and thicker dermis than the WT littermates (P < 0.0001 and P = 0.001 respectively, Supplementary Figure S2). Treatment with DMBA/TPA induced a substantial increase (P < 0.001) and approximate doubling in both dermal and epidermal thickness in both genotypes; both epidermis (P = 0.0103) and dermis (not significant) being apparently slightly thicker in the WT mice (the analysis was performed only from areas devoid of papillomas) (Supplementary Figure S2).

**Figure 3:**
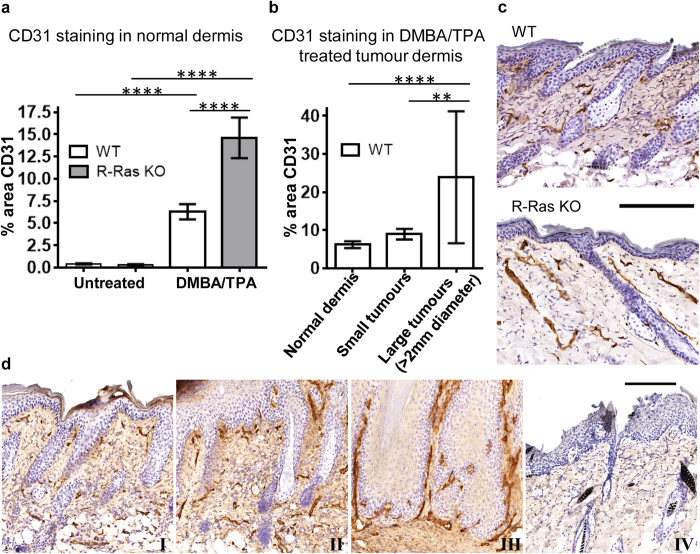
Deficiency of R-Ras leads to increased angiogenic response in the dermis after DMBA/TPA treatment. WT and R-Ras KO littermates were subjected to DMBA/TPA-induced skin carcinogenesis as described in methods. Skin samples were collected, fixed and processed for IHC. (**a**) The percentage area of dermis with microvasculature was determined by immunohistochemical staining for CD31 of 4% paraformaldehyde fixed, paraffin embedded sections of back skin (WT: n = 5; R-Ras KO: n = 4; 6 independent measurements per animal). Quantitative analysis of blood vessel density in dermis was performed by Spectrum digital pathology system/Image Scope analysis software as described in methods. The data were Log (10) transformed to fit a normal distribution and statistically analysed via GraphPad Prism 6. The values are shown as mean ± 95% confidence interval. Using a standard two-sided unpaired T-test, after DMBA/TPA treatment, the R-Ras KO mice have significantly more blood vessels in dermis than the WT mice (P < 0.0001, ****) despite showing almost no tumour formation. (**b**) The area of dermal staining for CD31 was analysed in DMBA/TPA-treated WT mice with tumours (n = 5). The dermis beneath large tumours (>2 mm) had significantly more blood vessels than the dermis beneath small tumours (<2 mm) (P = 0.0036, **) and the normal dermis (P < 0.0001, ****). There was not enough R-Ras KO tumour histology data for statistical analysis due to the lack of tumourigenesis in those animals. (**c**) Representative CD31 staining for blood vessels in DMBA/TPA-treated skin is shown for the WT and R-Ras KO animals. Despite having almost no detectable tumours, mice lacking R-Ras show an increased number of blood vessels in their skin after the DMBA/TPA-treatment. (**d**) The number of blood vessels is increased beneath skin tumours during DMBA/TPA-induced carcinogenesis in the WT animals. Representative images of CD31 staining are shown from a region of skin (I) with no visible tumour formation, (II) with a small tumour and (III) with a large tumour. (IV) The DMBA/TPA-treated skin sample from a WT mouse was stained with class-matched mouse IgG as a specificity control. Black bar in images represents 200 μm.

As the availability of vascular supply is a limiting factor for tumour growth and it has been shown that tumour xenografts are hypervascularised in R-Ras KO mice^9^,^10^, we examined the vasculature in the skin. There was no difference between the WT and R-Ras KO mice in the density of blood vessels in the normal, untreated skin as determined by CD31 immunohistological analysis (Fig. 3). However, treatment with DMBA/TPA induced a more than 10-fold increase in the vascular density of the skin in both genotypes (P < 0.0001, Fig. 3), and in line with previous studies^9^,^10^ the R-Ras KO mice developed significantly more blood vessels than the WT animals (P < 0.0001, Fig. 3a), by on average almost three-fold. Despite the fact that WT skin had significantly less blood vessels than the R-Ras KO mice after the DMBA/TPA-treatment, the tumour formation required vascular supply in the WT mice as evidenced by the increased number of blood vessels beneath the small and large tumours, the highest vascular density being beneath the large >2 mm tumours (P < 0.0001 non-tumour epidermis vs. large tumours and P = 0.0036 small vs. large tumours, Fig. 3b). Therefore the vascular supply of the skin did not account for the lack of tumour formation in the R-Ras KO animals, as they had significantly more blood vessels following DMBA/TPA treatment than the tumour-bearing WT mice (Fig. 3). Meaningful comparison of the blood vessel density between WT and R-Ras KO tumours was not possible because of the latter’s rarity and small size.

### R-Ras KO mice have decreased dermal cell proliferation and increased dermal cell apoptosis

**Figure 4:**
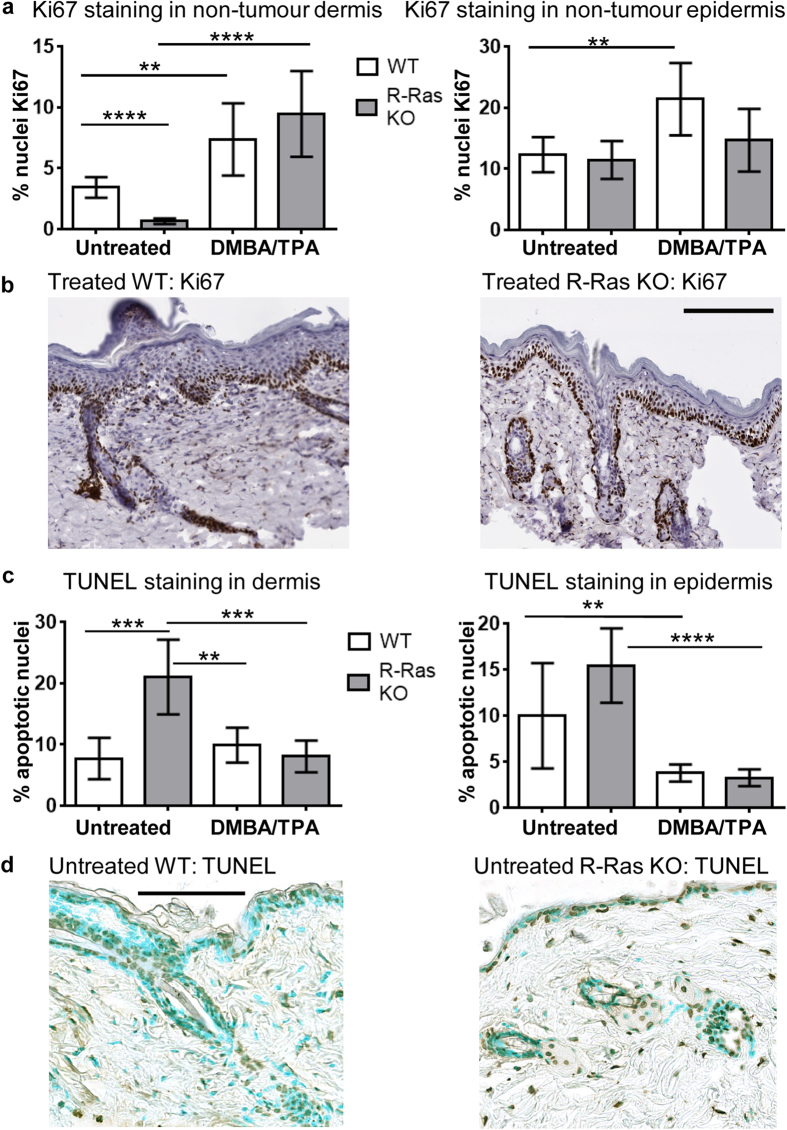
Cell proliferation is reduced and the number of apoptotic cells is increased in the normal dermis of R-Ras KO mice. WT and R-Ras KO mice were subjected to DMBA/TPA-induced skin carcinogenesis as described in methods. Skin samples were collected, fixed and processed for IHC staining of proliferating and apoptotic nuclei as described in methods. Quantitative digital pathology analyses of scanned slides were performed. Statistical analyses were performed with GraphPad Prism 6 software. Results are shown as mean ± 95% confidence intervals. The data were analysed by standard unpaired two-tailed T-tests. (**a**) Proliferating nuclei were stained by IHC with rat anti-Ki67 antibody, and the % of proliferating nuclei determined (WT: n = 5; R-Ras KO: n = 5; 3 independent measurements/animal). The WT mice have significantly more proliferating cells in both the dermis and the epidermis following DMBA/TPA treatment (standard unpaired two-tailed T-tests). The same phenomenon did not take place in R-Ras KO mice epidermis after DMBA/TPA treatment. Interestingly, prior to treatment the R-Ras KO mice had significantly fewer proliferating nuclei in their normal dermis than the WT animals (P < 0.0001, ****). (**b**) Representative Ki67 staining for proliferating cells in DMBA/TPA-treated skin is shown for the WT and R-Ras KO animals. Black bar in images represents 200 μm. (**c**) Immunohistochemical TUNEL staining for apoptotic nuclei was performed. TUNEL morphometry measurements of % epidermal and dermal apoptosis were taken from the untreated and DMBA/TPA-treated groups (WT: n = 5; R-Ras KO: n = 5; 4 independent measurements/animal). A couple of single outlying data points were identified by Grubbs’ test and excluded. Both the WT and the R-Ras KO mice have significantly fewer apoptotic cells in their normal epidermis after 19 weeks of DMBA/TPA treatment (P = 0.0097, ** and P < 0.0001, **** respectively). Untreated R-Ras KO mice have significantly more apoptotic cells in their normal dermis prior to DMBA/TPA treatment (P = 0.0003, ***), and significantly more apoptotic dermal cells than the WT mice either before (P = 0.0007, ***) or after (P = 0.0014, **) treatment. (**d**) Representative photograph of TUNEL staining in untreated WT and untreated R-Ras KO skin. Nuclei are stained turquoise, and apoptotic nuclei brown/black. The black bar in images represents 100 μm.

Although there was no difference in cell proliferation (as determined by Ki67) between the untreated WT mice epidermis and the untreated R-Ras KO mice epidermis, there were significantly more proliferating cells in the dermal part of the skin (excluding hair follicles) in untreated WT mice than in the untreated R-Ras KO mice (P < 0.0001, Fig. 4a). Treatment with DMBA/TPA induced a significant hyperproliferative response in the skin in both genotypes (Fig. 4a), but there were no statistically significant differences between the WT and R-Ras KO mice, either in epidermal or dermal cell proliferation following DMBA/TPA treatment. It was noted that (as commonly reported throughout the literature) the epidermis of tumours had the highest proportion of proliferating cells (Supplementary Figure S3), but in this respect there were no differences between WT tumours and the scarce and small R-Ras KO tumours. These data show that despite carcinogenesis taking place almost exclusively in the WT animals (rather than in the R-Ras KO mice) hardly any differences could be detected between the two genotypes in the proliferation of the epidermis where the tumours manifested. Because it has been reported that knockdown of R-Ras in gastric cancer epithelial cells enhances cell death^11^, we measured apoptosis in the skin of WT and R-Ras KO mice via TUNEL staining. Both the WT and R-Ras KO mice were found to have significantly more apoptotic cells in the normal epidermis (P = 0.0097 and P < 0.0001 respectively) before the DMBA/TPA-treatment than after (Fig. 4c), but no differences could be detected between the genotypes. Although there was no difference in the percentage of dermal apoptotic cells between the phenotypes after the DMBA/TPA treatment, significantly more apoptotic dermal cells were found in the untreated skin of the R-Ras KO mice than in the untreated WT mice (P = 0.0007) (Fig. 4c).

### Investigation of cell signalling

We decided to investigate the possible mechanism behind the skin tumourigenesis resistance phenotype in the DMBA/TPA treated R-Ras KO mice by studying signalling pathways associated with Ras.

Akt has been implicated in driving tumour formation in numerous cancers, including the DMBA/TPA-model^14^, because its activation can block apoptosis, and thereby promote cell survival^15^. Unexpectedly, at the 19^th^ week end point of the experiment, the phosphorylated Akt protein levels were dramatically reduced in response to DMBA/TPA-treatment and the reduction in the activation of Akt took place in an identical fashion in both genotypes (P = 0.0003 for WT and P = 0.0059 for R-Ras KO, Supplementary Figure S4a). A markedly low level of Akt phosphorylation in DMBA/TPA-induced papillomas (compared to carcinomas) has previously been reported^14^.

Ras GTPases activate cell proliferation via activation of the MAPK pathway^16^. First, we looked at the signalling events upstream of Ras, observing that Src (the phosphorylation of which is inhibited by R-Ras^17^) might be phosphorylated in slightly higher levels in untreated WT skin than in R-Ras KO skin, but the slight difference did not reach statistical significance (Supplementary Figure S4b). All studied MAPKs downstream of Ras (i.e. MEK1/2, p42 Erk1 and p44 Erk2) showed significant activation in similar fashion by the growth signal induced by the DMBA/TPA treatment in R-Ras KO and WT skin (Supplementary Figure S4c). However, no significant differences in their activation levels between the WT and R-Ras KO animals were detected (Supplementary Figure S4c). These results did not prompt us to study their role more thoroughly.

Rac1 has an essential role in tumour cell proliferation and survival^18^. It has been previously shown that Rac1 is crucial for Ras-dependent tumour formation in the same DMBA/TPA-skin carcinogenesis-model as employed here^18^. Furthermore, it was shown very recently that R-Ras is needed for Rac1 activation^19^,^20^. No significant changes in Rac1 expression were detected between the WT and R-Ras KO mice either before or after the DMBA/TPA-treatment (Supplementary Figure S4d). Phospho-Rac was undetectable in skin (data not shown), possibly because of its activation being dependent upon a lack of cell adhesion during cell migration^21^. Thus, this data implies that R-Ras-dependent tumour formation in the epidermis does not involve Rac1.

### R-Ras KO mice have an attenuated inflammatory response to DMBA/TPA treatment

**Figure 5:**
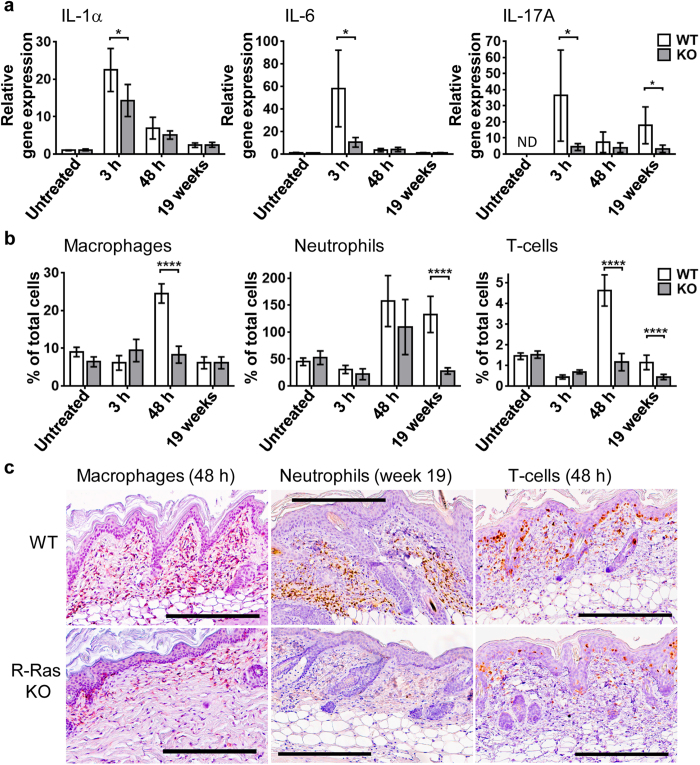
The inflammatory response to DMBA/TPA treatment is attenuated in the skin of R-Ras KO mice. WT and R-Ras KO mice were subjected to DMBA/TPA-induced skin carcinogenesis as described in methods. Skin samples were collected from untreated mice and from mice sacrificed at 3 h and 48 h after the second TPA application, and after 19 weeks of treatment (twice weekly). The skin samples were processed either for IHC or qPCR analysis as described in methods. Results are shown as mean ± 95% confidence intervals. Data were analysed by normality tests and unpaired two-tailed T-tests, if needed with Welch’s correction (GraphPad Prism 6). (**a**) qPCR analysis of relative gene expression of the cytokines *IL-1α*, *IL-6* and *IL-17A* in untreated and treated WT and R-Ras KO skin. R-Ras KO mice show 3 h post 2^nd^ TPA treatment significantly lower gene expression for *IL-1α* (P = 0.0176, *) and *IL-6* (P = 0.0125, *) than WT mice. *IL-17A* gene expression is significantly reduced in R-Ras KO animals at 3 h post 2^nd^ TPA treatment (P = 0.0322, *) and after 19 weeks of TPA treatment (P = 0.0189, *). These data are normally distributed. Animal numbers: untreated: n = 4 per strain, 3 h post 2^nd^ TPA treatment: n = 8 per strain, 48 h post 2^nd^ TPA treatment: n = 9 per strain, 19 weeks: n = 8 for WT and n = 6 for R-Ras KO. ND means not detected. (**b**) Skin sections were IHC stained for markers for dermal macrophages (F4/80), dermal neutrophils (neutrophil elastase), or epidermal and dermal T-cells (CD3), as described in methods (WT n = 6; R-Ras KO n = 6). Quantitative analysis of scanned slides were performed as described in methods (3 independent measurements per skin section, two skin sections per animal, excluding tumours). Data is expressed as % of total nuclei. Neutrophil and T-cell data are mostly normally distributed, but all becomes normally distributed if Log10 transformed. Macrophage data is normally distributed. T-tests confirmed highly significant differences between the WT and R-Ras KO mice as indicated (P < 0.0001, ****). (**c**) Representative photographs of leukocyte staining in WT and R-Ras KO skin. Nuclei are stained blue, and leukocytes brown. The black bar in images represents 300 μm.

It is an established fact that tumourigenesis in the DMBA/TPA model is highly dependent upon the induction of acute inflammation^22^. It has also recently been published that the R-Ras KO mouse has a reduced inflammatory response to experimental autoimmune encephalomyelitis due to increased tolerance in its immune system^23^. As our cellular transformation-focused investigations yielded no clue to the mechanism of resistance of the R-Ras KO mouse to DMBA/TPA-induced tumourigenesis, we decided to investigate if the R-Ras KO mouse had an attenuated skin inflammatory response to DMBA/TPA treatment. We measured the number of skin T-cells and infiltrating macrophages and neutrophils (via quantitative immunohistochemical analysis), as well as the gene expression of *IL-1α*, *IL-6* and *IL-17A* in the skin (via qPCR analysis) at a variety of different time points following the initiation of DMBA/TPA treatment (3 h and 48 h post second TPA treatment, and after 19 weeks of twice-weekly TPA treatment). These cytokines were selected for study because, not only are they proinflammatory, but IL-17A in particular is known to be pro-tumourigenic in the DMBA/TPA model^24^,^25^ and to work synergistically with IL-6^26^,^27^,^28^. At 3 h after the second TPA treatment, the R-Ras KO mice showed significantly lower levels of gene expression for *IL-1α*, *IL-17A* and *IL-6* compared to WT (P = 0.0176, P = 0.032 and P = 0.0125 respectively, Fig. 5a). Gene expression of *IL-17A* and *IL-6* in the R-Ras KO mice remained low at all of the time points tested. The level of *IL-17A* expression in the R-Ras KO mice remained low even at the 19-week time point whilst it was significantly elevated in the WT mice (P = 0.0189, Fig. 5a). The leukocyte count data showed the lack of a significant increase in skin macrophage infiltration or T-cells in the R-Ras KO mice at any time point, with neutrophil infiltration in the R-Ras KO mice only apparently elevated at 48 h after the second TPA treatment (Fig. 5b). In contrast, in the WT mice there was a clear increase in T-cell and macrophage numbers, which was significantly greater than in the R-Ras KO mice at the 48-h time point (P < 0.0001 for each, Fig. 5b). At the 19-week time point the WT mice had significantly more T-cells and especially more neutrophils than the R-Ras KO mice (P < 0.0001 for each, Fig. 5b). When comparing the timing of the inflammatory differences between the WT and R-Ras KO mice, it was noted that the lack of cytokine expression in the R-Ras KO mice was actually most evident preceding, not following, leukocyte infiltration (extravasation) (Fig. 5). The leukocyte count data in Fig. 5b is expressed as % of total nuclei rather than cells per mm^2^ as either format gave very similar results (Supplementary Figure S5a), but the former is more reliable in histological areas that are slightly damaged. A measure of total cells per mm^2^ in all groups revealed that there were no differences in total cell density per mm^2^ dermis between the WT and R-Ras KO mice, either before or after treatment (Supplementary Figure S5b).

## Discussion

In summary, our study indicated an important role of the small GTPase R-Ras in epidermal hyperplasia and tumour induction in a skin carcinogenesis-tumour model. This is striking and unexpected, as R-Ras has previously been associated with cellular quiescence and the inhibition of cellular proliferation and invasion, but not with the promotion of cancer^2^,^5^.

Our finding is also novel in the sense that we could not detect any R-Ras protein expression in the epidermis, from where the tumours arose, only in the dermal blood vessels (Fig. 2). This result is in line with a previous report where it was proven that normal epithelial tissues do not express R-Ras protein^10^ as well as with the fact that R-Ras itself possesses very little transformative activity in cells. However, we could not see an induction of R-Ras expression by tumour or normal epidermal cells in response to chemically induced carcinogenesis, which is opposite to the finding that gastric and cervical epithelial tumours express R-Ras abundantly^11^. It has recently been published that R-Ras expression and activation in breast cancer cells makes the cells generally less oncogenic *in vitro*, and the expression of activated R-Ras protein is actually significantly lower in breast tumours than in the surrounding normal tissue^5^. Combined with the findings from our study, this raises the possibility that strong R-Ras expression in surrounding stroma or neighboring tissues could be more relevant than expression in the tumour cells themselves in the context of tumour initiation. Thus, a key issue is whether the strong stromal R-Ras expression identified around breast carcinomas^5^ contributes to tumour induction and formation in breast cancer in the same fashion as the R-Ras expression in the blood vessels in our study.

Our findings could imply that epidermal papilloma formation in WT mice (expressing R-Ras) occurs by mechanisms similar to those responsible for the development of gastrointestinal polyposis^29^. In gastrointestinal polyposis, it is the loss of the Lkb1 gene from surrounding mesenchymal stroma (not the tumour) that causes tumour formation due to reduced TGFβ production by stromal cells that support the tumour, and defective TGFβ-signalling in epithelial cells, coinciding with their subsequent enhanced proliferation^29^.

Interestingly, it has recently been found that a lack of R-Ras has anti-inflammatory effects^23^,^30^. The DMBA/TPA skin tumourigenesis model is dependent upon proinflammatory responses, as continuous TPA treatment provides a constant inflammatory stimulus, and the deletion of various proinflammatory genes prevents the tumourigenesis^22^. As inflammation and cancer have been found to be highly linked, cancer-related inflammation is proposed to be one of the hallmarks of cancer^31^,^32^. Thus, an attenuated inflammatory response identified in R-Ras KO mice in response to DMBA/TPA treatment might explain their resistance to skin tumourigenesis. Indeed, our results (Fig. 5) support this hypothesis. We found a reduction of *IL-1α* mRNA and an even clearer reduction of *IL-6* and *IL-17A* gene expression in the DMBA/TPA-treated R-Ras KO skin. The lack of *IL-17A* gene expression in the R-Ras KO mice persisted at the 19-week time point when tumours and elevated *IL-17A* mRNA levels were detected in the WT mice. The lack of IL-17A is of particular interest because IL-17A has been proven to be strongly pro-tumourigenic in the DMBA/TPA model^24^,^25^, and also to be a critical cytokine in combination with IL-6 for the inhibition of CD4^+^ T-cell immunosurveillance and apoptosis, and for the enhanced recruitment of macrophages and neutrophils^26^,^27^,^28^, the latter of which are pro-tumourigenic in this and other cancer models^33^,^34^. A reduced number of IL-17-producing CD4^+^ T-cells in R-Ras KO mice has been reported previously^23^.

We also found a lack of leukocyte infiltration in the treated R-Ras KO skin, which is of significance because both CD4^+^ T-cells^35^ and neutrophils^33^,^34^ are pro-tumourigenic in the DMBA/TPA model and certain macrophages support tumour growth^36^,^37^. This lack of leukocyte extravasation could hypothetically be explained by the lack of R-Ras in the KO endothelium, which would be likely to inhibit migratory leukocyte adhesion and transmigration^38^,^39^,^40^. However, the greatest deficiency in inflammatory cytokine gene expression in the R-Ras KO was observed at the time point (3 h) prior to extravasation (48 h) in response to DMBA/TPA treatment (Fig. 5). This suggests that the cells of the immune system already present within the untreated R-Ras KO skin have an impaired potential to express inflammatory cytokines in response to TPA. This is in line with the reported observation that the R-Ras KO mouse has an increased level of tolerogenic immune cells and a lack of proinflammatory cells^23^,^30^. It is difficult to postulate exactly what deficiencies of the inflammatory response in the R-Ras KO mouse are responsible for its resistance to DMBA/TPA induced tumourigenesis, or the mechanism by which the loss of R-Ras expression has led to these deficiencies. Indeed, the striking absence of the normally increased macrophage and T-cell numbers in the R-Ras KO skin at 48 h after the second TPA treatment is hard to explain, because neutrophil numbers were still able to increase at the same time point (Fig. 5). R-ras has been shown to regulate the proinflammatory phospholipase Cε (PLCε)^41^, which plays a crucial role in TPA-induced skin inflammation^42^. Like R-Ras KO mice, PLCε KO mice are also resistant to tumour formation in the DMBA/TPA model^42^. However, PLCε is expressed in keratinocytes and dermal fibroblasts, whilst R-Ras is not.

Here, we have shown that R-Ras KO mouse skin undergoes excessive angiogenesis in response to DMBA/TPA treatment (Fig. 3), and it has been published that such excessive angiogenesis in the R-Ras KO mouse is accompanied by excessive vascular leakage^2^,^9^,^10^. Although it is published that vascular leakage and leukocyte infiltration are not interdependent events^43^, they are often observed together and enhanced vascular permeability increases leukocyte chemotaxis^44^. This further underlines the unusualness of the attenuated inflammatory responses observed in the R-Ras KO mouse.

Despite recent efforts to characterise the R-Ras signalling pathway, the down-stream effectors of R-Ras are still poorly understood^2^,^45^. Our results further emphasise the need to explore unconventional mechanisms of action for R-Ras in tumour induction in the skin. In particular the mechanism by which R-Ras affects the function of the immune system and the inflammatory response requires further study. Additional carcinogenesis models and the generation of conditional R-Ras knockout or conditional R-Ras over-expressing mice would be useful. Such studies could shed further light upon the potential role of R-Ras in tumourigenesis as well as how inflammation leads to the development of cancer.

## Methods

### Mice

Homozygous knockout mice deficient for R-Ras expression, which were generated by an insertion into the *R-Ras* gene region between exons 4 and 5 on chromosome 7 (R-Ras KO mice) have been described previously^10^, and were obtained from the laboratory of Masanobu Komatsu (Sanford-Burnham Medical Research Institute at Lake Nona, Orlando, FL. USA). Before any experiments, R-Ras heterozygous mice were backcrossed eight generations with C57BL/6 strain (Harlan) to obtain R-Ras expressing (wild-type, WT) and R-Ras KO strain in the same background genetic strain of mice (littermates). Then homozygous R-Ras KO animals were bred. The genotype was determined in each animal by PCR^10^ and the lack of R-Ras expression was later confirmed by standard immunoblotting techniques. Mice were fed with standard laboratory pellets and water *ad libitum*. All animal experiments were performed in accordance with protocols approved by the National Animal Ethics Committee of Finland.

### Skin tumour induction

R-Ras KO and C57BL/6 WT mice were treated with DMBA and TPA to induce skin tumours^13^. In brief, the backs of 8-week-old mice were shaved and 24 h later 50 μg DMBA (7,12-Dimethylbenz[a]anthracene) (Sigma, Dorset, UK) in 200 μl acetone was applied topically on the shaved area of the dorsal skin. After a week, the back skin of the mice was treated twice a week with 5 μg TPA (12-*O*-tetradecanoylphorbol-13-acetate) (Sigma) in 200 μl acetone for 19 weeks. Tumours (1 mm in diameter or larger) were counted twice a week. The fur excluding tumours was carefully shaved every two weeks.

### Immunohistochemical (IHC) and TUNEL staining

Samples of back skin from sacrificed, shaved control mice or mice at week 19 of the tumour induction experiment were collected and fixed with 4% paraformaldehyde and embedded in paraffin according to standard protocols. Hematoxylin/eosin staining and DAB immunohistochemical staining (IHC) was performed on 6 μm thick paraffin sections. The following primary antibodies were used for IHC (according to the manufacturer’s instructions): LS-C147992 rabbit anti-R-Ras (Lifespan Biosciences, Seattle, WA, USA), M7249 TEC-3 rat anti-Ki67 and A0452 rabbit anti-CD3 (DakoCytomation, Glostrup, Denmark), 550274 rat anti-CD31 (BD Pharminogen, Oxford, UK), 68672 rabbit anti-neutrophil elastase (AbCam, Cambridge, UK) and MF48000 BM8 rat anti-F4/80 (Life Technologies Ltd., Paisley, UK). The blocking reagents used for IHC were S2O23 REAL and S0809 Antibody Diluent (Dako). In the case of blocking prior to CD3 or neutrophil elastase staining, G9023 goat serum or A4503 BSA (Sigma) were used respectively, at 5% in PBS. The horseradish peroxidase (HRP) conjugated secondary antibody reagents used were: PO448 goat anti-rabbit (Dako), 414311F anti-rat Histofine (Nichirei Bio, Tokyo, Japan) and for neutrophil elastase staining RMR622 Rabbit on Rodent (Biocare Medical, Concord, CA, USA). XMF963 XM-Factor (Biocare) was used to block before secondary staining with Rabbit on Rodent. Peroxidase reactive chromogens used were K3465 DAB (DAKO) and RAEC810 Romulin AEC (BioCare). Immunohistochemical TUNEL staining for apoptotic nuclei was performed using the K403-50 TUNEL IHC Kit (Biovision, Milpitas, CA, USA) with Methyl Green nuclear counter stain, according to the manufacturer’s instructions.

### Quantitative analysis of immunostaining and histochemical staining

All slides were scanned using the Aperio ScanScope® CS and XT systems (Aperio Technologies Inc., California, USA)^46^. Slides were viewed and analysed remotely using desktop personal computers employing the web-based ImageScope™ viewer. The Spectrum digital pathology system analysis algorithm package and Image Scope analysis software (version 9; Aperio Technologies Inc.) were applied to quantify immunohistochemical signal. These algorithms calculate the area of positive staining, the average positive intensity (optical density), as well as the percentage of weak (1+), medium (2+), and strong (3+) positive staining^46^. All quantified histochemical analyses (Ki-67, CD31, TUNEL) were performed according to the protocols used to established these algorithms for each respective staining^46^,^47^.

### Preparation of skin lysates, western blot analysis and densitometry

Samples of back skin from sacrificed, shaved control mice or mice at week 19 of the tumour induction protocol were removed with a scalpel on ice. Skin was immediately frozen with liquid nitrogen and later lysed in 10 μl of cold RIPA buffer per 1 mg of tissue with added c*O*mplete protease inhibitor (Roche Applied Science, Penzberg, Germany) and Thermo HALT phosphatase inhibitor (Life Technologies Ltd., Paisley, UK). The tissues were homogenised using CK14 beads and a homogeniser (Precellys, Yvelines, France), and the protein concentration of the supernatants measured by Invitrogen Qubit (Life Technologies). From each sample 100 μg of protein was loaded per well of an Invitrogen NuPAGE 4–12% gradient gel (Life Technologies) and electrophoresed alongside Invitrogen Magic Mark and See Blue standards (Life Technologies), and electroblotted according to the manufacturer’s instructions. For detection of specific proteins by immunoblotting the following primary antibodies were used (according to the manufacturer’s instructions): rabbit polyclonals against total Akt (pan), Phospho-Akt (Ser473), Phospho-Src (Tyr416), Cdc42, Phospho-Rac1/cdc42 (Ser71), Rac1/2/3, Phospho-p44/42 MAPK (Erk1/2) (Thr202/Tyr204), Phospho-MEK1/2 (Ser217/221), total p44/42 MAPK (Erk1/2), and R-Ras were from Cell Signaling Technology (Danvers, MA, USA). Rabbit anti-β-actin was from Millipore (Espoo, Finland), and Goat anti-GAPDH was from AbCam (Cambridge, UK). Secondary reagents used were horseradish peroxidase-coupled anti-rabbit from Cell Signaling Technology. Western blot images were captured without saturation via ImageQuant (GE Lifesciences, Amersham, UK) and quantified by densitometry using ImageJ software, where β-actin or GAPDH was used to normalise for different protein loading amounts.

### Extraction of RNA

Samples of back skin were harvested and snap frozen in liquid nitrogen, stored at −80 °C, and preserved in Ambion RNALater ICE Frozen Tissue Transition Solution (Life Technologies). The mRNA was then extracted using Invitrogen Trizol Reagent (Life Technologies) according to the manufacturer’s instructions, with bead homogenisation performed via Precellys’ 2 ml tubes and homogeniser at 4 °C. After the addition of chloroform and centrifugation, the aqueous phase was transferred to RNeasy columns (Qiagen, Hilden, Germany) and mRNA purification continued according to the manufacturer’s instructions. The concentration and quality of RNA was determined by Nanodrop A260 measurement and by “bleach gel” analysis^48^.

### Quantitative PCR (qPCR) analysis

Total skin RNA was converted to cDNA by reverse transcription using the Thermo Maxima First Strand cDNA Synthesis Kit for RT-qPCR (Life Technologies) according to the manufacturer’s instructions, at 55 °C for 30 min at concentrations not exceeding 150 ng/μl. 50 ng cDNA (for IL-1α, IL-6, reference genes HPRT and TBP), or 100 ng cDNA (for IL-17A, HPRT and TBP) were used for qPCR analysis, which was performed in white 96-well Multiply PCR plates (Sarstedt, Nümbrecht, Germany) in a 7500 Real-Time PCR System (Life Technologies). Samples were measured in triplicates. The qPCR reaction was done in a reaction volume of 25 μl with the Thermo Maxima SYBR Green/ROX qPCR Master Mix (2x) (Life Technologies) according to the manufacturer’s instructions with primers for IL-1α, IL-6, IL-17A (all 300 nM), and the reference genes HPRT (250 nM) and TBP (300 nM). See Supplementary Table S1 for primer details. As negative controls, no-template and no-reverse transcriptase controls were also included (which were herein negative). The thermal cycler profile for all primer sets was: 2 min 50 °C, 10 min 95 °C, 40 × (15 sec 95 °C, 30 sec 59 °C, 32 sec 72 °C data collection). At the end of thermal program, a melting curve analysis was always performed to check for unspecific PCR products (which herein never occurred). Raw fluorescence data were analysed with the LinRegPCR program (version 2014.8, free download http://LinRegPCR.HFRC.nl). The LinRegPCR program is based on the methods and procedures described by Ruijter *et al.*, 2009^49^. It was chosen because it belongs to one of the most reliable methods for analysing quantitative qPCR data^50^,^51^. Cq (quantification cycle) values of over 35 were considered as “not detected” (ND). LinRegPCR calculates the starting concentration N0 per sample (well) expressed as arbitrary fluorescence units, which can be seen as the non-normalised expression values. LinRegPCR includes in its calculation the mean PCR efficiency per amplicon (N0 = threshold/(mean amplicon efficiency^Cq^ )). N0 values were used for the calculation of relative gene expression normalised to the geometric mean^52^ of the 2 reference genes’ N0 values (geometric mean of N0 values of replicates of gene of interest divided by geometric mean of N0 values of reference genes). In addition, for better visualisation the relative gene expression is shown relative to the condition “WT untreated” (set to 1) or multiplied by a factor of 1000 (only for IL-17A).

### Statistical analysis

Mean averages are shown with 95% confidence intervals. All data were analysed to determine if it was normally distributed (D’Agostino & Pearson omnibus and Shapiro-Wilk normality tests). Significance at a given time point was calculated by two-tailed Student’s *t*-test for normally distributed data. An alpha level less than 0.05 was considered significant. Survival plot data were analysed by log-rank (Mantel-Cox) test and non-normally distributed time course data were analysed by non-linear regression. Prism 6 (GraphPad Software, La Jolla California, USA) was used for a majority of the analyses and STATA 13 (StataCorp LP, College Station, Texas, USA:) statistical analysis software was used for non-linear regression analysis, as indicated.

## Additional Information

**How to cite this article**: May, U. *et al.* Resistance of R-Ras knockout mice to skin tumor induction. *Sci. Rep.* **5**, 11663; doi: 10.1038/srep11663 (2015).

## Supplementary Material

Supplementary Information
