# Supplementary material for: Resistance of R-Ras knockout mice to skin tumour induction
Source: Sci Rep. 2015 Jul 2;5:11663. doi: 10.1038/srep11663 (PMC4488886; doi:10.1038/srep11663)
Supplement: Supplementary Information [file srep11663-s1.doc]

**Supplementary information:**

**Resistance of R-Ras knockout mice to skin tumour induction**

Ulrike May, Stuart Prince, Maria Vähätupa, Anni M Laitinen, Katriina Nieminen,

Hannele Uusitalo-Järvinen & Tero AH Järvinen

**Supplementary Figure S1**


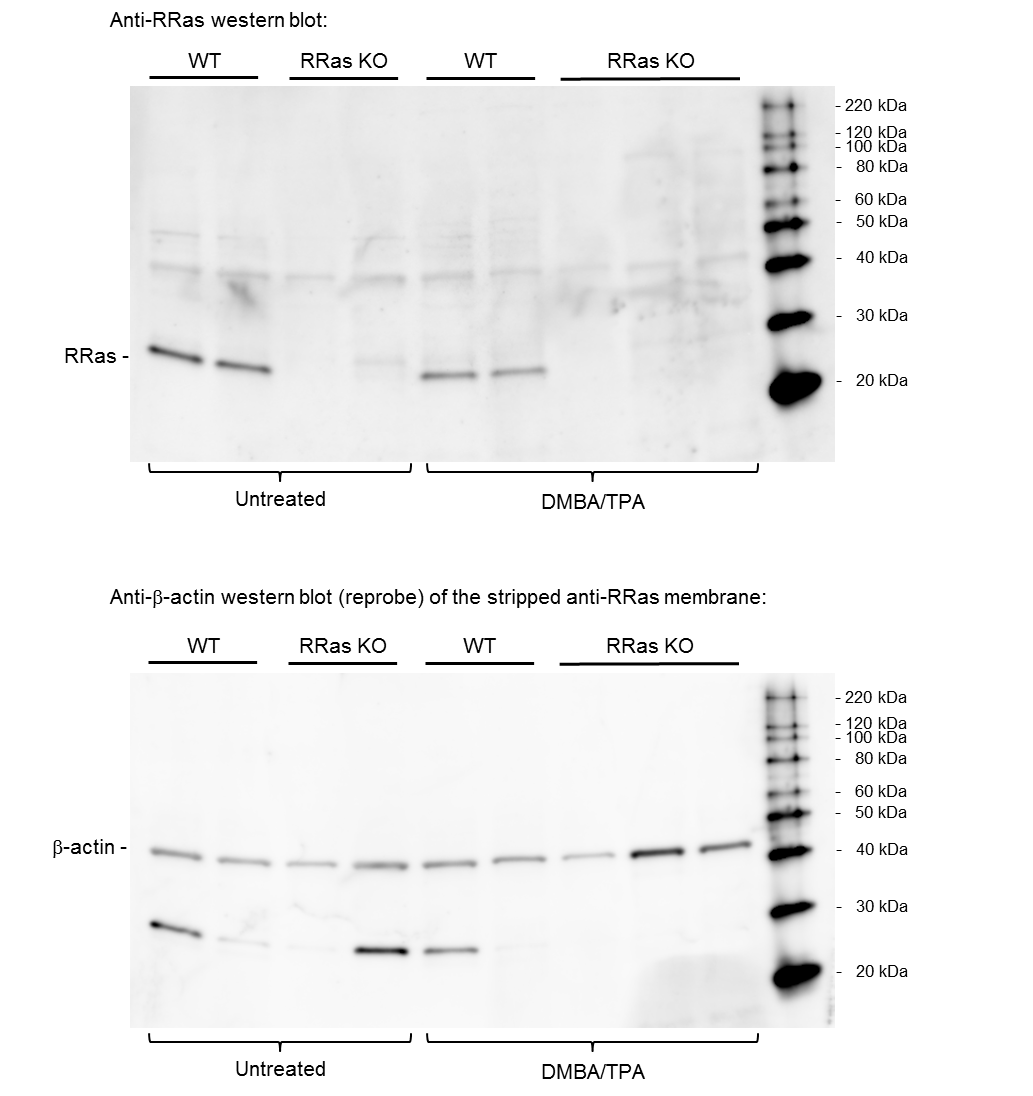


**Supplementary Figure S1. Western blot analysis of mouse skins.** Both untreated and DMBA/TPA-treated WT and RRas KO mouse skins were collected, homogenised and lysed in RIPA buffer for western blot analysis, and R-Ras expression detected as described in the methods section, followed by stripping and reprobing for β-actin as a loading control.


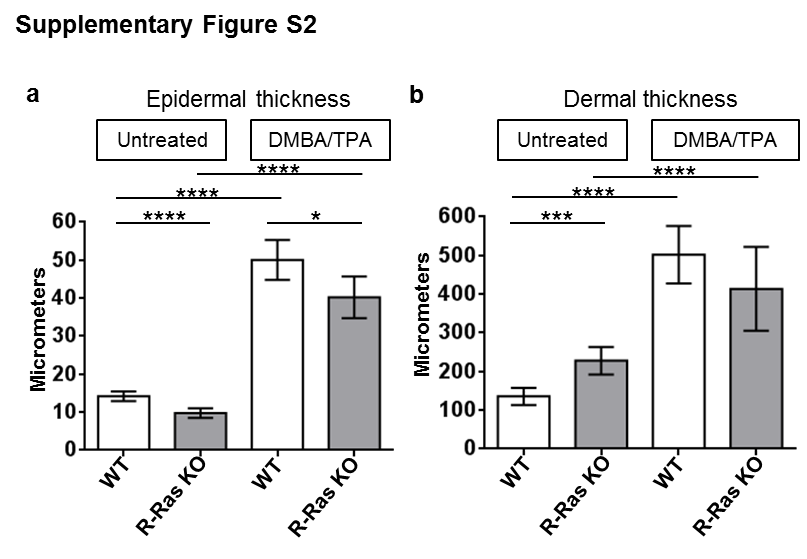


**Supplementary Figure S2. Thickness of normal epidermis and dermis.** (**a**) Measurements of epidermal thickness of non-tumour regions of skin from paraffin sections in triplicates from 8 Wild-type (WT) and 9 R-Ras knockout mice (R-Ras KO). Using standard unpaired two-tailed T-tests, the WT mice were found to have a slightly thicker epidermis than the R-Ras KO mice after 19 weeks of DMBA/TPA treatment (P=0.0103 *), but the difference is more apparent before DMBA/TPA treatment (P<0.0001 ****). (**b**) Measurements of dermal thickness were taken (in triplicates) from 5 WT and 4 R-Ras KO mice. The data from the DMBA/TPA treated groups is not significantly different. Prior to DMBA/TPA treatment, the dermis of the R-Ras KO mice appears slightly thicker (P=0.001 ***). The results are expressed as mean ± 95 % confidence intervals.

**Supplementary Figure S3**


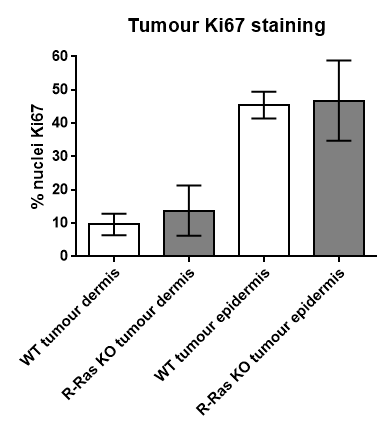


**Supplementary Figure S3. Ki67 staining of tumours.** WT and R-Ras KO mice were subjected to DMBA/TPA-induced skin carcinogenesis as described in methods. Skin samples were collected, fixed and processed for IHC staining of proliferating nuclei with rat anti-Ki67 antibody with nuclear counterstain. The % of nuclei positive for Ki67 in the dermis and epidermis of tumours was determined. Results are shown as mean ± 95% confidence intervals. Statistical analyses were performed with GraphPad Prism 6 software. The data were analysed by standard unpaired two-tailed T-tests. Despite the scarcity and small size of R-Ras KO tumours, enough data was collectable for a meaningful comparison (WT: n=14; R-Ras KO: n=5). There is no significant difference in % Ki67 nuclei between WT and R-Ras KO tumours. There is no significant difference between tumour and non-tumour DMBA/TPA-treated dermal % Ki67 nuclei (in comparison to the data in Figure 4a). Both WT and R-Ras KO tumour epidermis have significantly more % Ki67 nuclei than non-tumour DMBA/TPA-treated epidermis (in comparison to the data in Figure 4a, P<0.0001).

**Supplementary Figure S4a-d**


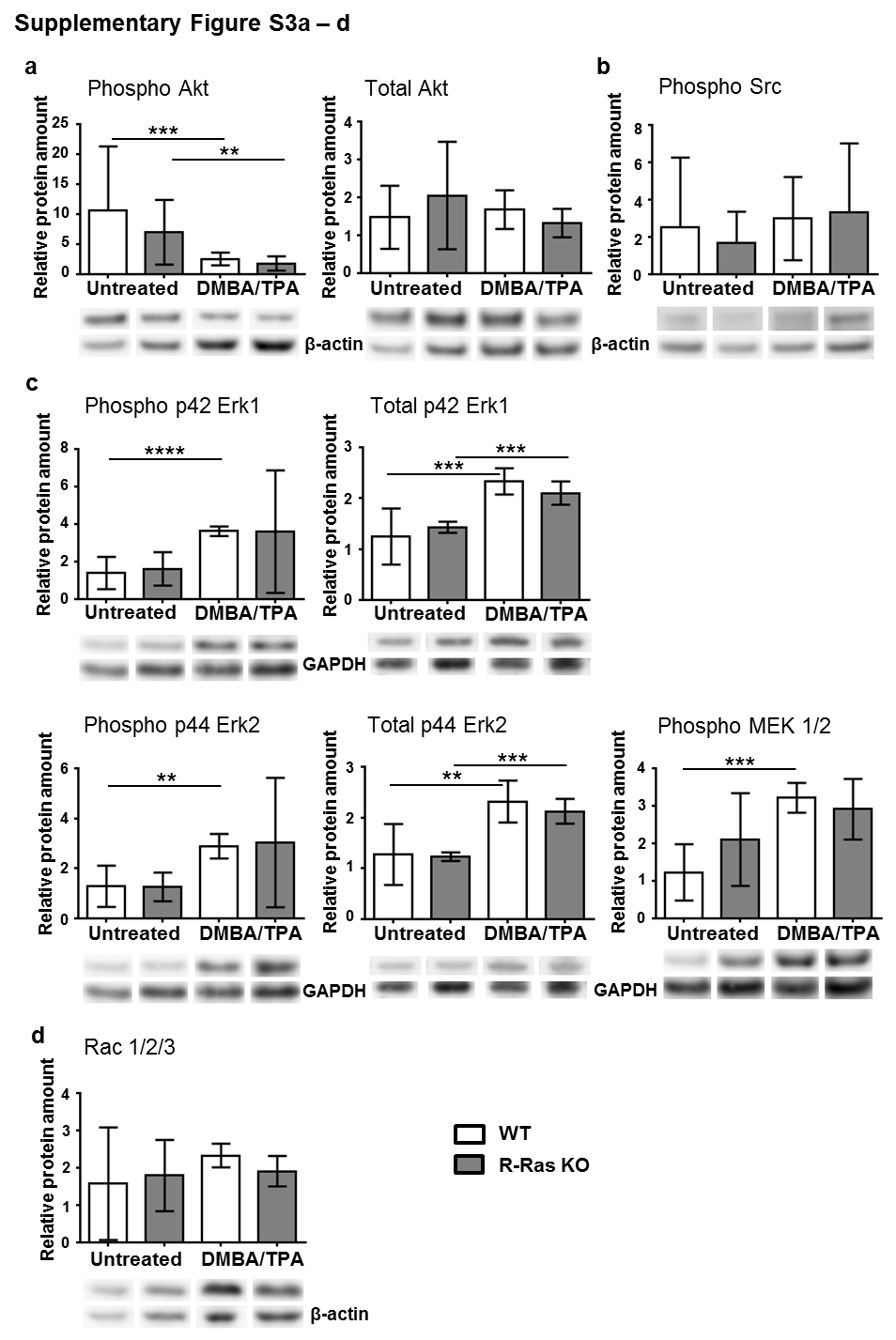


**Supplementary Figure S4a to d. Loss of R-Ras does not affect known R-Ras associated signalling pathways in R-Ras KO mouse skin.** Untreated skin samples from 3 WT and 3 R-Ras KO mice, and 19-week DMBA/TPA treated skin samples from 4 WT and 4 R-Ras KO mice were analysed by western blotting as described in the methods section. All independent tissue samples per mouse strain/condition were run on the same blot. The blots were stained with antibodies against phosphorylated and non-phosphorylated forms of signalling partners known to be involved in R-Ras signalling: total Akt, Phospho-Akt, Phospho-Src, Phospho-Rac1/cdc42, Rac1/2/3, Phospho-p44/42 MAPK (Erk1/2), Phospho-MEK1/2 and total p44/42 MAPK (Erk1/2). The blots were stripped and reprobed for β-actin or GAPDH to normalise for loading. The data was analysed by densitometry relative to the loading control (LoCtr) to calculate the relative protein amount (ImageJ), and Student’s T-test. Representative examples from western blot pictures (cropped) are displayed under every graph (protein of interest in the upper and loading control in the lower panel). No significant difference in cell signaling between WT and R-Ras KO skin was detected.

**(S4a) Akt signalling in total skin.** Akt phosphorylation significantly decreases following 19 weeks of DMBA/TPA treatment (WT P=0.0003 ***, KO P=0.0059 **). LoCtr was β-actin.

**(S4b) Src signalling in total skin.** No significant differences were observed in Src phosphorylation. LoCtr was β-actin.

**(S4c) MAPK signalling in total skin.** Following DMBA/TPA treatment: p42 Erk1 phosphorylation increases (WT P<0.001 ****, KO P=0.166); total p42 Erk1 expression increases (WT P=0.0006 ***, KO P=0.0007 ***); p44 Erk2 phosphorylation increases (WT P=0.0012 **, KO P=0.1274); total p44 expression increases (WT P=0.0029 **, KO P=0.0002 ***); and MEK 1/2 phosphorylation increases (WT P=0.0002 ***, KO not significant). LoCtr was GAPDH.

**(S4d) Rac signaling in total skin.** There were no significant differences in Rac 1/2/3 expression (shown). Western blots for phospho-Rac1/cdc42 (Ser71) were also performed but no phospho-Rac1 was detected in skin (data not shown). LoCtr was β-actin.

**Supplementary Figure S5**

**Supplementary Figure S5a-b**. **Total cell density in the dermis remains constant regardless of genotype, DMBA/TPA treatment, and analysis format. (a)** Neutrophil counts both in cells per mm^2^ and % total cells format were determined as described in methods and compared. The results are essentially the same for the data displayed in both formats, with the difference being that the intra-group variance is slightly lower and the distribution of data (whether log transformed or not) is more normal for the % total cells format. **(b)** Two skin sections from each untreated or DMBA/TPA treated WT or R-Ras KO animal (n=5 animals per group) were analyzed by HE staining and digital pathology analysis to determine the number of cells per mm^2^ in each of three large gated non-tumour dermal regions per skin section. There were no differences in the cell density per mm^2^ between groups, despite the infiltration of leukocytes and the replication of cells, suggesting that cells remain distributed evenly throughout the experiment.

**Supplementary Table S1**

**Supplementary Table S1. Primers used for SYBR Green qPCR.** All primer pairs have been designed to span exons to amplify only mRNA but not genomic DNA. The primers don’t bind to known SNP positions.
